# Shorter duration of venetoclax administration to 14 days has same efficacy and better safety profile in treatment of acute myeloid leukemia

**DOI:** 10.1007/s00277-023-05102-y

**Published:** 2023-01-16

**Authors:** Masayuki Aiba, Akio Shigematsu, Toma Suzuki, Takuto Miyagishima

**Affiliations:** Kushiro Rosai Hospital, Kushiro, Hokkaido Japan

**Keywords:** Venetoclax, Short duration, Acute myeloid leukemia, Myelosuppression

## Abstract

Venetoclax (VEN) is now widely used in the treatment of acute myelogenous leukemia (AML) in elderly patients who are not eligible for intensive remission induction therapy. Prolonged myelosuppression, increased incidence of infection, and long duration of hospital stay were major concerns for VEN treatment cases, and we thought that shortening the duration of VEN administration during induction therapy might solve these problems. Thirteen newly diagnosed AML patients who underwent VEN+azacitidine (AZA) induction therapy from March 2021 to June 2022 at Kushiro Rosai Hospital were analyzed retrospectively. The median age was 79 (range, 68–86) years, and 8 of the patients (61.5%) were classified as high risk according to the ELN 2017 risk stratification. Eight patients received VEN for 14 days (VEN14 group), and 5 patients received VEN for 28 days (VEN28 group). The composite complete remission (CRc) rate was 76.9% in total, and the CRc rates in the VEN14 and VEN28 groups were almost the same (75.0% and 80.0%, respectively). The median overall survival (OS) was not reached in the VEN14 group and was 254 days in the VEN28 group. The median event-free survival (EFS) was not reached in the VEN14 group and was 178 days in the VEN28 group. The VEN14 group might have a possibility to reduce febrile neutropenia (37.5% vs. 80%) and reduce the duration of hospital stay (median, 21.5 vs. 31 days) compared with the VEN28 group. VEN14 produced the same CRc rate and survival rate, safer profile, and shorter duration of hospital stay than VEN28.

## Introduction

Acute myelogenous leukemia (AML) mainly affects the elderly, and the standard treatment is combination chemotherapy. However, elderly patients are less suitable for intensive combination chemotherapy due to their age and comorbidities, and less intense chemotherapy such as low-dose cytarabine or azacitidine (AZA) monotherapy has been used, but the complete remission (CR) rate with AZA monotherapy is less than 30% with poor prognosis [1].

Venetoclax (VEN) in combination with AZA (VEN+AZA) has been reported to provide a high CR rate, 66.4%, in the treatment of AML in the VIALE-A trial [2]. Therefore, in Japan, VEN obtained insurance coverage for AML in 2021 and is now widely used in the treatment of AML in elderly patients who are not eligible for intensive induction therapy. In the VIALE-A trial, VEN was continued until day 28 of each cycle, but high incidences of myelosuppression and febrile neutropenia (FN), which are noteworthy side effects, were reported.

Most of the AML patients at our hospital are elderly and have poor performance status (PS), and it is important to avoid progressive disuse and death due to adverse event. Therefore, we thought that reducing the duration of VEN administration to 14 days in cycle 1 of the treatment might achieve a better safety profile and shorten the hospitalization period during induction therapy, with the same efficacy in the treatment of AML.

### Patients and method

This study included retrospectively all patients with newly diagnosed AML who underwent induction therapy with VEN+AZA therapy for at least 1 cycle from March 2021 to June 2022 at the Kushiro Rosai Hospital. Poor cytogenetic risk was defined as genetic abnormality classified in adverse risk category according to the European Leukemia Net 2017 criteria [3].

VEN was administered in combination with intravenous or subcutaneous AZA 75 mg/m^2^ daily for 7 days. The dose of VEN was 100 mg on day 1 and 200 mg on day 2; on day 3, the target dose of 400 mg was reached and continued according to the intake period. We classified patients into VEN14 and VEN28 groups based on the duration of VEN administration. In the VEN14 group, VEN was taken for the first 14 days and then stopped until the start of the next cycle. In the VEN28 group, VEN was taken for 28 days. Shortening of the duration of VEN administration after cycle 1 was permitted according to the degree of myelosuppression in the VEN28 group. To promote recovery of blood count, the start of the next cycle of VEN+AZA could be postponed when cytopenia greater than grade 4 occurred after clearance of leukemia cells from the bone marrow. The dose of VEN was adjusted based on concomitant medication with CYP3A4 inhibitors such as voriconazole or itraconazole. Filgrastim was not used to promote neutrophil count recovery.

Responses were evaluated as per standardized criteria. Complete remission (CR) was defined as an absolute neutrophil count of more than 1000 cells per cubic millimeter, a platelet count of more than 100,000 per cubic millimeter, red cell transfusion independence, and bone marrow with less than 5% blasts. Composite remission rate (CRc) was defined as marrow CR with or without hematologic recovery (CR and CRi), and hematologic recovery was defined as an absolute neutrophil count of more than 1000 cells per cubic millimeter, a platelet count of more than 100,000 per cubic millimeter, and red cell transfusion independence. The date of relapse was defined as the date of the first bone marrow test after CRc consistent with disease relapse. We used Wilms tumor gene (WT1) in peripheral blood (PB) as a marker of minimal residual disease (MRD), and the upper normal value for it was set at 50 copies/μg RNA, with a sensitivity of 10^−4^ to 10^−5^ [4]. Transfusion independence was defined as absence of a red cell or platelet transfusion for at least 56 days between the first and last day of treatment. Adverse events were summarized according to the National Cancer Institute Common Terminology Criteria for Adverse Events Version 5.0. Duration of hospital stay was defined as the period from the start of VEN treatment to discharge for any cause.

The clinical data cutoff date was August 9, 2022. We used the EZR version 1.55 to conduct statistical analysis [5]. Overall survival (OS) and event-free survival (EFS) were calculated using the Kaplan-Meier method. OS was defined as the period from the start of VEN treatment to the last follow-up or death from any cause, and EFS was defined as the period from the start of VEN treatment to disease relapse, treatment cessation, or any cause of death. We used the log-rank test to compare survival curves and gray test to compare cumulative incidence of hematologic recovery. We used Fisher’s exact test to compare ratios between groups, such as the rate for incidence of FN. We used a nonparametric test (Mann-Whitney *U* test) to compare the period of admission and period of FN between treatment groups. This trial was conducted in accordance with the Declaration of Helsinki, and the protocol and related documents were approved by the ethics committees at Kushiro Rosai Hospital.

## Results

Table 1 shows the main baseline characteristics of the study population at diagnosis and prior to VEN treatment. A total of 13 patients were identified, 10 men and 3 women, with a median age of 79 years (range, 72 to 86). Eight patients were in the VEN14 group and five patients were in the VEN28 group. The median follow-up period was 141 days (range, 28 to 176) in the VEN14 group and 192 days (range, 30 to 295) in the VEN28 group. 87.5% of the patients in the VEN14 group and 20.0% of the patients in the VEN28 group had baseline neutropenia greater than grade 3. 62.5% of the patients in the VEN14 group and 60.0% of the patients in the VEN28 group had AML with poor cytogenetic risk.

**Table 1:** Baseline characteristics according to the duration of VEN

	Total (*N* = 13)	VEN14 group (*N* = 8)	VEN28 group (*N* = 5)
Age, median (range)	79 (72–86)	80 (72–83)	79 (75–86)
WT1 in PB, median (range)	8300 (50–87,000)	7200 (50–79,000)	8300 (250–87,000)
ECOG PS ≥ 2, *n* (%)	3 (23.1)	1 (12.5)	2 (40.0)
Poor cytogenetic risk, *n* (%)	8 (61.5)	5 (62.5)	3 (60.0)
Cytopenia ≥ grade 3, *n* (%)			
Neutropenia	8 (61.5)	7 (87.5)	1 (20.0)
Thrombocytopenia	5 (38.5)	1 (12.5)	4 (80.0)

Abbreviations; WT1, Wilms Tumor gene; PB, Peripheral Blood; PS, Performance Status

ECOG PS scores range from 0 to 5, with 0 indicating no symptoms and higher scores indicating greater disability. Cytogenetic risk category was classified based on the ELN 2017 risk stratification. Cytopenia was graded according to the Common Terminology Criteria for Adverse Events

**Fig 1:**
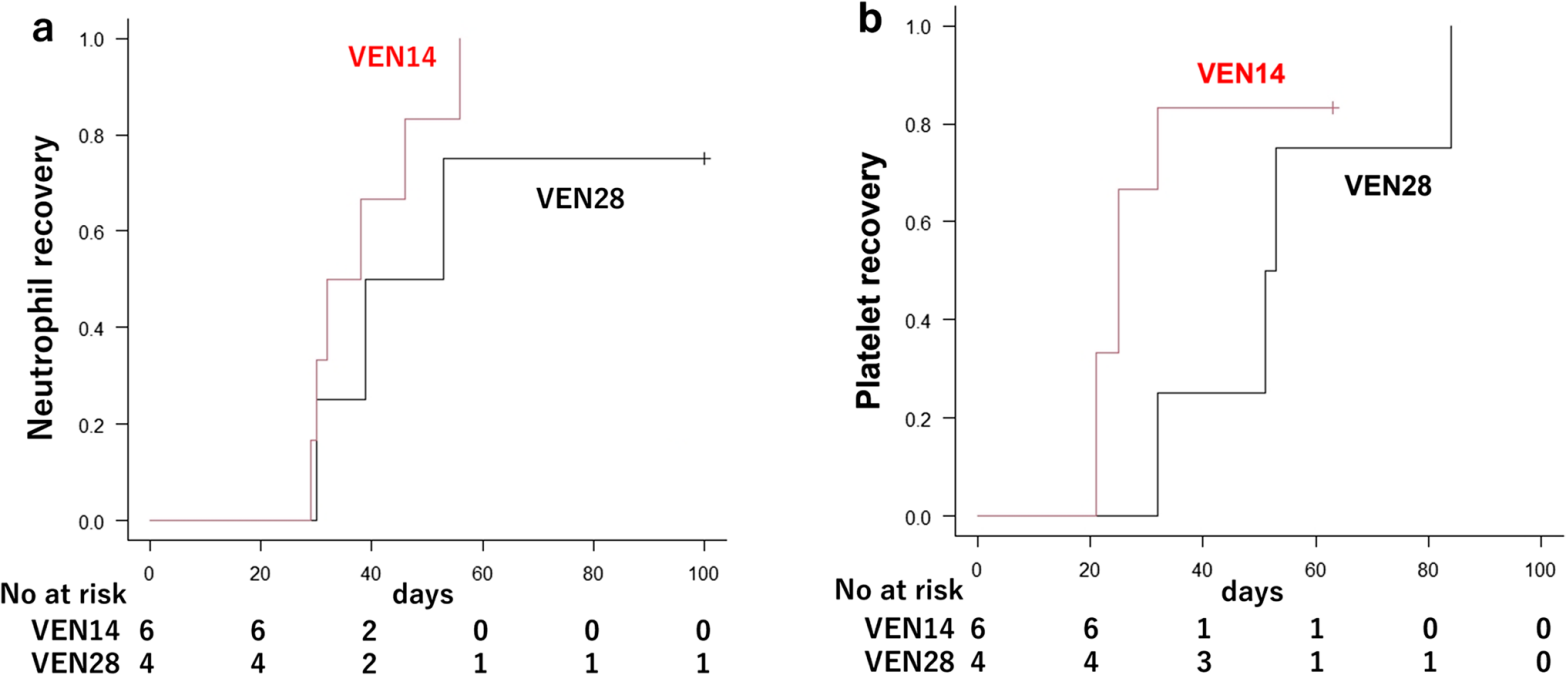
Cummulative incidence of hematologic recovery in patients who achieved CRc after VEN+AZA therapy. **a** Neutrophil recovery was defined as absolute neutrophil count of more than 1000 cells per cubic millimeter. **b** Platelet recovery was defined as platelet count of more than 100,000 per cubic millimeter

**Fig 2:**
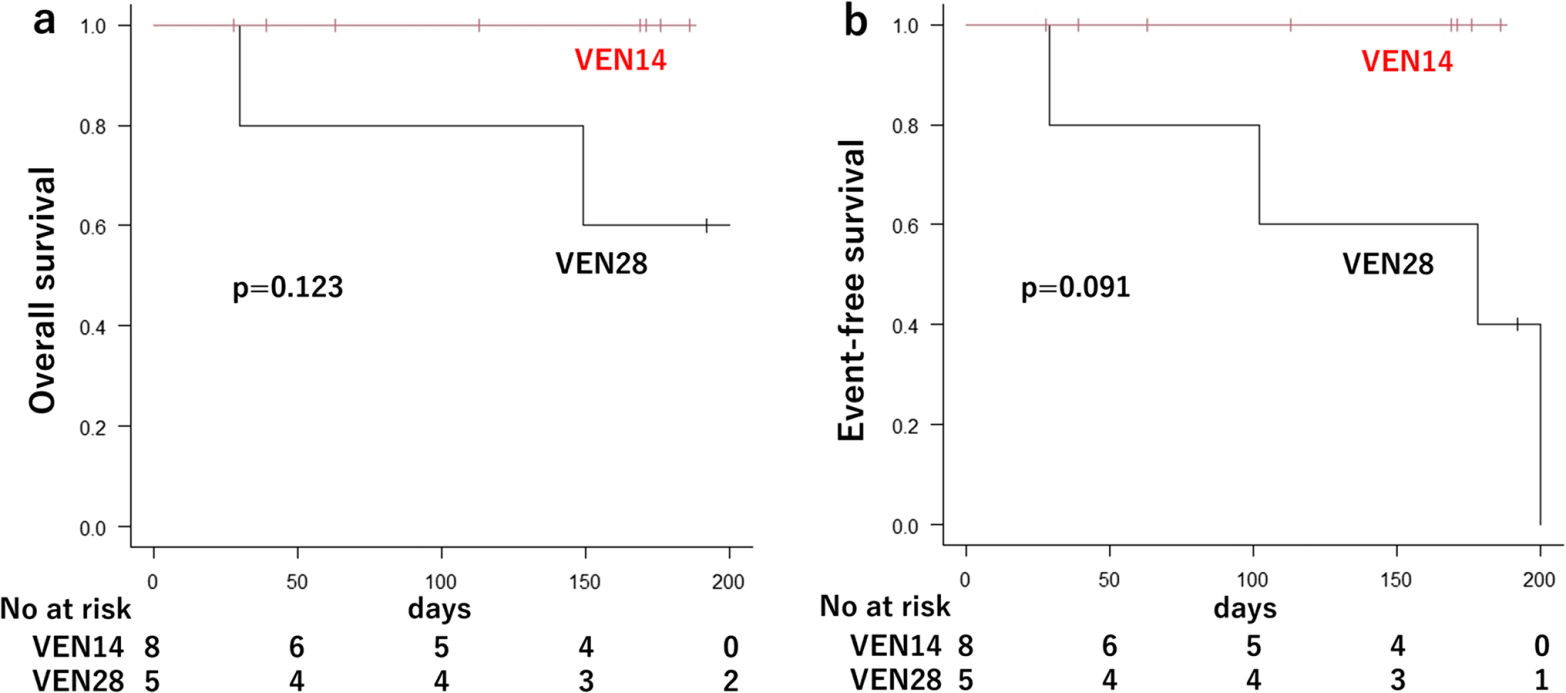
**a** Overall survival and **b** event-free survival in each group. The distributions were estimated for each group with the use of the Kaplan-Meier method. The data included are subject to a cutoff date of August 9, 2022, and the median follow-up was 169 days (range, 28–192 days)

Table 2 shows the effectiveness of the treatment in the VEN14 and VEN28 groups. Responses for 7.7% of the patients were not available (death or lack of data). Total CRc, CR, and CRi rates over the entire observation period were respectively 76.9%, 46.2%, and 30.8%. Overall CR rate was 50.0% in the VEN14 group and 40.0% in the VEN28 group. Overall CRc rate was 75.0% in the VEN14 group and 80.0% in the VEN28 group. CRc rate before initiation of cycle 2 was 62.5% in the VEN14 group and 60.0% in the VEN28 group. Among patients with intermediate cytogenetic risk, overall CRc rate was 66.7% in the VEN14 group and 100% in the VEN28 group. Among patients with poor cytogenetic risk, overall CRc rate was 80.0% in the VEN14 group and 66.7% in the VEN28 group. The median time from VEN+AZA treatment to CRc was 26 days (range, 22 to 113) in the VEN14 group and 31 days (range, 20 to 84) in the VEN28 group. Figure 1 shows a graph for cumulative recovery of neutrophil and platelet counts in patients who achieved CRc. The median days from VEN+AZA therapy to neutrophil count recovery was 38 days in the VEN14 group and 39 days in the VEN28 group, and median days from VEN+AZA therapy to platelet recovery was 25 days in the VEN14 group and 52 days in the VEN28 group. The median OS was not reached [NR] (95% confidence interval [CI], NR to NR) in the VEN14 group and 254 days (95% CI, 30 to NR) in the VEN28 group. The median EFS was NR (95% CI, NR to NR) in the VEN14 group and 178 days (95% CI, 29 to NR) in the VEN28 group. Estimated OS and EFS rate at 6 months were respectively 100% (95% CI, not available [NA] to NA) and 100.0% (95% CI, NA to NA) in the VEN14 group, and 60.0% (95% CI, 12.6 to 88.2) and 40.0% (95% CI, 5.2 to 75.3) in the VEN28 group (Fig. 2). The percentage of patients achieving WT1-negative status seemed to be higher in the VEN14 group (50.0% vs. 20.0%, *p* = 0.565), and estimated median EFS was better in patients who achieved WT1 negativity than those who did not (NR vs. 178 days, *p* = 0.087). Red cell transfusion independence occurred in 87.5% of the patients in the VEN14 group and 60.0% of the patients in the VEN28 group, and platelet transfusion independence occurred in 87.5% of the patients in the VEN14 group and 80.0% of the patients in the VEN28 group.

**Table 2:** Efficacy of VEN+AZA according to the duration of VEN

	VEN14 group (*N* = 8)	VEN28 group (*N* = 5)
Overall CR, *n* (%)	4 (50.0)	2 (40.0)
CR before cycle 2, *n* (%)	4 (50.0)	2 (40.0)
Overall CRc, *n* (%)	6 (75.0)	4 (80.0)
CRc before cycle 2, *n* (%)	5 (62.5)	3 (60.0)
Days from VEN to CRc, median (range)	26 (22–113)	31 (20–84)
WT1 reduction before cycle 2, median (range)	−7152 (−74,500 to +300)	−35,105 (−86,050 to −1540)
Overall WT1 negativity, *n* (%)	4 (50.0)	1 (20.0)
Transfusion independence, *n* (%)		
Red cells	7 (87.5)	3 (60.0)
Platelets	7 (87.5)	4 (80.0)

WT1 reduction refers to WT1-mRNA reduction in PB (copy/μg RNA). Achievement of WT1 refers to the WT1-mRNA in PB reduction to less than 50

The safety profile (Table 3) reveals that FN occurred in 37.5% of the patients in the VEN14 group and 80.0% of the patients in the VEN28 group. The median duration of FN was 1.0 day in the VEN14 group and 3.5 days in the VEN28 group (*p* = 0.593). The median duration of hospital stay was 21.5 days in the VEN14 group and 31 days in the VEN28 group (*p* = 0.378). Four of the 13 patients died during follow-up, five due to the exacerbation of AML and three due to pneumonia (Table 4). The overall mortality rate was 0.0% in the VEN14 group and 80.0% in the VEN28 group.

**Table 3:** Safety of VEN+AZA according to the duration of VEN administration

	VEN14 group (*N* = 8)	VEN28 group (*N* = 5)
FN, *n* (%)	3 (37.5)	4 (80.0)
During cycle 1	3 (37.5)	4 (80.0)
After cycle 2	1 (12.5)	0 (0.0)
Days of neutrophil count < 500, median (range)	30 (0–56)	28 (8–228)
Days of FN, median (range)	1 (1–10)	3.5 (2–5)
Days of hospital stay, median (range)	21.5 (12–41)	31 (17–44)

Abbreviation: *FN* febrile neutropenia; FN was defined as a fever of 37.5 °C or higher with neutrophils less than 500/μL

**Table 4:** Mortality according to the duration of VEN administration

	VEN14 group (*N* = 8)	VEN28 group (*N* = 5)
Overall death, *n* (%)	0 (0.0)	4 (80.0)
Death due to pneumonia, *n* (%)	0 (0.0)	1 (20.0)
Death due to disease progression, *n* (%)	0 (0.0)	3 (60.0)

## Discussion

This is the first clinical report showing the results of shortening VEN administration to 14 days from cycle 1 in AML patients treated with VEN+AZA. The CRc rate in the VEN14 group was not inferior to that in the VEN28 group. Gangat et al. proposed that in frail elderly patients, a shortened course of VEN to 14 days starting cycle 1 be considered [6], but the data on safety and efficacy are still unknown.

For the efficacy, the overall CRc rate in our study was 76.9% in total, 75.0% in the VEN14 group and 80.0% in the VEN28 group, which was almost equal to that in the VIALE-A trial (66.4%) [2]. Considering the cytogenetics of AML, the proportion of patients with poor cytogenetic risk is reported to increase in elderly patients [7], and patients with poor risk have poorer OS than patients with favorable risk [8]. Compared with patients studied in the VIALE-A trial, in our study the median age was higher (79 vs. 76 years old), and the percentage of patients with poor cytogenetic risk was also higher (61.5% vs. 36.4%). These data imply that shortening VEN administration to 14 days may be as effective as the standard protocol even in the poor-risk AML patients in real-world settings.

In our study, WT1 was used as a MRD marker, and the rate of total WT1 negativity was higher in the VEN14 group than in the VEN28 group (50.0% and 20.0%, respectively), while WT1 reduction in PB in the VEN14 group after cycle 1 was less than that in the VEN28 group (median, −7152 vs. −35,105, *p* = 0.368). Estimated EFS was better in patients who achieved WT1 negativity than those who did not (median, NR vs. 178 days, *p* = 0.087). The recent meta-analysis showed that achievement of MRD negativity is associated with superior disease-free survival (DFS) and OS in patients with AML [9, 10], and OS was similar among patients who achieved CRc with an MRD-negative response after cycle 1 and thereafter [10]. Ujj et al. proposed that disappearance of WT1 positivity during chemotherapy had a favorable effect on survival and no difference was observed between the survivals of WT1-positive subgroups that expressed moderate or high levels of WT1 [11], which means that achieving WT1 negativity is important in the treatment of AML. In our study, none of the patients in the VEN28 group could start cycle 2 of VEN+AZA as scheduled due to prolonged myelosuppression, whereas 50.0% of the patients in the VEN14 group could, which may have resulted in higher overall WT1 negativity in the VEN14 group.

Regarding the safety data, the incidence of FN was lower in the VEN14 group than in the VEN28 group (37.5% vs. 80.0%). Overall mortality and non-relapsed mortality were also lower in the VEN14 group than in the VEN28 group and VIALE-A trial (0% vs. 80.0% vs. 56.2% and 0% vs. 20.0% vs. 26.9%, respectively) [2]. Arora et al. showed that FN and new infections developed in 27% and 25% of patients during cycle 1 of VEN-based therapy, and infectious complications during cycle 1 of VEN-based therapy led to poorer survival outcomes [12]. Therefore, we considered that VEN14 would lead to less incidence of FN, which may lead to shorter hospital stays. However, the duration of neutropenia less than 500/μL was longer in the VEN14 group (30 vs. 28 days), but this may be explained by the fact that more patients in the VEN14 group had neutropenia greater than grade 3 at the start of treatment than did patients in the VEN28 group (87.5% vs. 20.0%). Our study suggests that by shortening the duration of VEN administration to 14 days, patients with severe neutropenia prior to treatment can be safely treated without increasing FN and mortality rate.

In conclusion, our retrospective study suggests that shortening the duration of VEN administration to 14 days may reduce the risk of complications and be as effective as 28-day administration in the treatment of AML. Our study was limited by its small sample size and short duration of follow-up, so we could not demonstrate statistically significant differences. Further studies are needed to determine more precisely the effect and safety of shortening the duration of VEN administration to 14 days.
